# Antimicrobial Activity of Essential Oils in Vapor Phase In Vitro and Its Application in Combination with Lactic Acid to Improve Chicken Breast Shelf Life

**DOI:** 10.3390/foods12224127

**Published:** 2023-11-14

**Authors:** Jovany Fortino Rivera de la Cruz, Laura Inés Schelegueda, Sofía Belén Delcarlo, María Fernanda Gliemmo, Carmen Adriana Campos

**Affiliations:** 1 Departamento de Industrias, Facultad de Ciencias Exactas y Naturales, Universidad de Buenos Aires, Buenos Aires 1428, Argentina; jovany.fortino@gmail.com (J.F.R.d.l.C.); laura.schelegueda@gmail.com (L.I.S.); sofiabdelcarlo@gmail.com (S.B.D.); mfg@di.fcen.uba.ar (M.F.G.); 2 Instituto de Tecnología de Alimentos y Procesos Químicos (ITAPROQ), CONICET—Universidad de Buenos Aires, Buenos Aires 1428, Argentina

**Keywords:** chicken breast, essential oils in vapor phase, lactic acid, oregano, garlic, mustard

## Abstract

The effect of essential oils (EOs) incorporated in their vapor phase combined with lactic acid immersion pretreatment was studied on fresh refrigerated chicken breast shelf life. Among the several EOs assayed, the in vitro results obtained from the vapor diffusion test allowed mustard, oregano, and garlic EOs to be selected due to their higher antimicrobial activity. In addition, it was possible to determine the EO minimum inhibitory concentrations against *Pseudomonas aeruginosa* and *Escherichia coli* and to identify EO binary mixtures showing synergistic or additive effects. Based on the obtained results, a ternary mixture constituted by 0.073, 0.292, and 0.146 µL/mL of headspace of mustard, oregano, and garlic, respectively, was proposed for its application to chicken breasts. The ternary mixture inhibitory action was confirmed in vitro against *P. aeruginosa* and *E. coli*. Furthermore, the presence of numerous compounds with recognized antimicrobial and antioxidant activity was found in its volatile phase through gas chromatography. When applying an EO mixture in its vapor phase in combination with 1.0% *v*/*v* of lactic acid immersion pretreatment on refrigerated chicken breast, a decrease in mesophilic microorganisms’ growth rate as well as in lipid oxidation was observed. Moreover, in a preliminary sensory test, the treated chicken breast was found to be acceptable to consumers and showed no significant differences compared to untreated chicken. In conclusion, the combined use of lactic acid immersion and EOs in their vapor phase was an effective alternative to increase chicken breast shelf life.

## 1. Introduction

Poultry meat has great importance for food safety as well as huge economic value worldwide since it represents approximately 40% of the total meat production and 37% of meat exports [1]. Among poultry meat, chicken consumption has increased due to its low cost of production [2]. However, the extension of its shelf life is a major concern for the industry since its high pH, moisture, and protein content promote the growth of spoilage and pathogenic microorganisms [3]. Furthermore, its high concentration of polyunsaturated fatty acids makes it prone to oxidative rancidity, resulting in the need for the incorporation of antioxidants into formulated products [4,5].

Consumers’ concerns over the use of synthetic chemical preservatives and the need to reduce the economic losses of spoiled foods have encouraged the search for natural preservation technologies applied in the context of hurdle technology. Among natural preservatives, organic acids, particularly lactic acid, were able to inhibit microbial growth and extend the shelf life of poultry meat [6,7]. Since it is obtained via fermentation and generally recognized as safe (GRAS), its use was allowed by The United States Food and Drug Administration [8]. As expected, lactic acid activity is enhanced when used in combination with other hurdles such as ultraviolet light (UV-C) treatment, modified atmosphere packaging, and other natural preservatives [9,10,11]. Regarding other natural preservatives, the use of essential oils (EOs) is promising since they exhibit antimicrobial, antifungal, virucidal, and antioxidant properties [12,13,14,15]. However, the direct use of EOs in food presents disadvantages because the complexity of food matrices (pH, proteins, lipids, sugars, fibers, etc.) attenuates the antimicrobial activity of these preservatives, thus requiring higher concentrations, which can exert a negative effect on the sensory properties of food. To overcome this disadvantage, EOs can be added as nanoemulsions into films or in the vapor phase of the packaging. Inclusion of thyme EO into chitosan composite films has been shown to extend the shelf life of chicken fillets, but the EOs increased the water vapor permeability of the film [13]. Furthermore, these films were not commercially available. The addition of EOs in the vapor phase was an alternative applied to control mold growth in beef jerky and Mexican corn tortillas [16,17] and bacterial growth in radish sprouts, alfalfa seeds, and hake fillets [18,19,20]. To the best of our knowledge, vapor-phase EOs have not been applied alone or in combination with lactic acid for the extension of chicken’s meat shelf life. From this perspective, the objective of this study was to select a combination of essential oils able to control bacterial growth with adequate sensory properties and study their use in combination with lactic acid to improve the shelf life of refrigerated chicken breast.

## 2. Materials and Methods

### 2.1. Essential Oils and Other Chemicals

The essential oils (EOs) tested were obtained from EUMA (Buenos Aires, Argentina), and they were lemongrass (*Cymbopogon citratus*), ginger (*Zingiber officinale*), tarragon (*Artemisia dracunculus*), basil (*Ocimum basilicum*), sage (*Salvia officinalis*), garlic (*Allium sativum*), mustard (*Brassica nigra*), rosemary (*Rosmarinus officinalis*), oregano (*Origanum vulgare*), coriander (*Coriandrum sativum*), thyme (*Thymus vulgaris*), and pepper (*Piper nigrum).* Dimethylsulfoxide (DMSO) and lactic acid were purchased from Biopack (Buenos Aires, Argentina), and trichloroacetic acid, thiobarbituric acid (TBA), and 1,1,3,3-tetraethoxy-propane were obtained from Sigma Aldrich (Saint Louis, MO, USA).

### 2.2. Bacterial Strains and Culture Conditions

*Pseudomonas aeruginosa* ATCC 9027, *Pseudomonas fluorescens* ATCC 49838, and *Lactobacillus plantarum* ATCC 8014 were used to represent spoilage microorganisms present in poultry meat. Similarly, *Listeria innocua* ATCC 33090 and *Escherichia coli* ATCC 35218 and 25922 were used as surrogates for pathogen *L. monocytogenes* and *E. coli* O157:H7, respectively. These strains were selected due to their similar characteristics to the pathogenic ones [21,22]. The Inocula used in all tests were made by growing twice every strain in trypticase soy broth for 24 h at 30 °C, and then the turbidity was adjusted to 0.5 McFarland units with peptone water (0.1% *w*/*v*) in a densimeter Densichek (Bioartis, Guipry, France), which is equivalent to 10^8^ Colony Forming Units (CFU) per mL.

### 2.3. Antimicrobial Activity of Essential Oils in Vapor Phase In Vitro

#### 2.3.1. Screening of Essential Oils’ Antimicrobial Activity

The antimicrobial activity of EOs in their vapor phase was individually evaluated against each bacterial strain using the methodology described by Goñi et al. [23]. Briefly, plates of 5.0 cm in diameter containing 15 mL of trypticase soy agar were surface inoculated with 500 µL of a 10^6^ CFU/mL inoculum of each bacterium. Then, sterile filter discs of 6 mm in diameter (Oxoid, Basingstoke, UK) were placed in the center of the plate lids, and 10 µL of each specific EO was dispensed; thus, only the vapor phase of the EO had contact with the agar surface. The concentration of the EOs in their vapor phase was 1.17 µL/mL of headspace (HS). This concentration was estimated by considering the complete volatilization of the 10 µL of EOs added to the plate HS, calculated as the difference between the total volume of the plate (21.24 mL) and the volume of solidified agar (12.74 mL). Plates without EO were used as the control. The plates were sealed with parafilm and incubated at 30 °C for 48 h. The antimicrobial activity of the EOs was evidenced by the presence of an inhibitory halo above the filter disc.

#### 2.3.2. Determination of the Minimal Inhibitory Concentration against *P. aeruginosa* and *E. coli*

The EOs showing the highest inhibitory activity in the vapor phase diffusion assay were selected for the evaluation of their minimal inhibitory concentration (MIC) against the growth of *P*. *aeruginosa* as representative of the spoilage biota and the pool of both *E. coli* strains as representative of the pathogenic biota. The MIC was determined using the vapor phase diffusion assay. For this purpose, EO was serially diluted two-fold in DMSO, testing concentrations in the range of 0.036 to 1.17 µL of EO/mL of HS. Then, 10 µL of each dilution was added to the sterile filter discs. Plates without EO and using only DMSO were used as the control. The MIC was determined as the concentration of the lowest dilution that generated a lack of visible bacterial growth on the agar surface.

#### 2.3.3. Determination of the Fractional Inhibitory Concentration Index of Essential Oil Binary Mixtures against *P. aeruginosa* and *E. coli*

The determination of the fractional inhibitory concentration index (FIC_i_) was carried out with binary mixtures of EOs, selected according to results obtained in the MIC evaluations. Mixtures of two EOs containing inhibitory and subinhibitory levels were tested against *P. aeruginosa* and *E. coli* following the Berenbaum design [24]. The different combinations evaluated are shown in Table 1. For the binary mixtures, the FIC_i_ was calculated using the following equation:

(1) $${\mathrm{FIC}}_{i}=\frac{{\mathrm{MIC}}_{A-B}}{{\mathrm{MIC}}_{A}}+\frac{{\mathrm{MIC}}_{B-A}}{{\mathrm{MIC}}_{B}}$$

where MIC_A-B_ is the MIC value of EO A in the presence of EO B. MIC_B-A_ is the MIC value of EO B in presence of EO A. MIC_A_ is the MIC value of EO A, and MCI_B_ is the MIC value of EO B. According to the results, if FIC_i_ ≤ 0.5, then the interaction is synergistic, if FIC_i_ is between 0.5 and 4.0, then the interaction is additive and if FIC_i_ > 4.0, then the interaction is antagonistic [25].

#### 2.3.4. Determination of the Effect of a Ternary Mixture against *P. aeruginosa* and *E. coli*

Based on the results, a ternary mixture containing 1/2 of the MIC of each EO was proposed for application to chicken breasts. As a first stage, its effect against both microorganisms was determined in vitro. The selection of the mixture was based on the requirement to apply the three EOs to inhibit a broad spectrum of microorganisms and on the fact that to observe an inhibitory effect in a food matrix, higher concentrations of antimicrobials than those determined in vitro are necessary [26,27].

### 2.4. Chemical Analysis of Essential Oils in Vapor Phase

The chemical compounds present in the HS of the selected ternary mixture were determined. For this purpose, a solid-phase microextraction fiber support (Bellefonte, PA, USA) coated with polydimethylsiloxane to absorb volatile compounds was used. Before the extraction, the support was held for 30 min at 250 °C in the gas chromatograph injection port to release impurities. The compounds were analyzed via gas chromatography coupled to a mass spectrum using a Perkin Elmer Clarus 500 GC-FID-MS system equipped with a single split–splitless injector. The methodology proposed by Delcarlo et al. [20] was used. The mass analysis covered a range of masses (*m*/*z*), from 40 to 300 Da. The compound identification was based on their retention indices relative to C8–C20 n-alkanes and compared with reference compounds (Vasana S.A., Villa Martelli, Argentina) or compounds previously identified in essential oils. The relative percentage distribution of each compound was calculated from the FID responses using computerized integration, assuming that all response factors were one.

### 2.5. Antimicrobial Activity of Essential Oils in Vapor Phase on Chicken Breast

#### 2.5.1. Chicken Breast Preparation

Skinless frozen chicken breasts were purchased in a commercial store (Buenos Aires, Argentina). The breasts were washed with chlorinated water (50 ppm) and then with sterile water and drained for 2 min. After that, they were cut into 3 cm long and 1 cm thick pieces and put into plastic containers (6.7 cm diameter and 4 cm high), whose lids had attached a 4 cm paper filter disc seeded with a selected EO ternary mixture. Some systems received an immersion in lactic acid for 5 min and were drained for 2 min as pretreatment. The systems evaluated were (i) a control chicken breast free of EOs and lactic acid immersion (C-C); (ii) a chicken breast exposed to the selected EO ternary mixture (C-EO); (iii) a chicken breast immersed in 1.0% *v*/*v* lactic acid solution (C-LA); and (iv) a chicken breast immersed in 1.0% *v*/*v* lactic acid solution and exposed to the selected EO ternary mixture (C-LA-EO). All systems were stored at 4.0 ± 0.1 °C, and samples were withdrawn after 0, 2, 3, 5 and 7 days of storage.

#### 2.5.2. Microbiological Analysis

The aliquots of each system were homogenized with peptone water, serially diluted, and poured-plated on Plate Count Agar (PCA) to enumerate the mesophilic aerobic microorganisms. The colonies were counted after incubation at 30 °C for 48 h, and the results were expressed as CFU per gram of chicken meat (CFU/g).

#### 2.5.3. Physicochemical Analysis

The pH was determined through a pH meter attached to a meat-piercing electrode (Fisher, Waltham, MA, USA). Lipid oxidation was determined through the thiobarbituric acid index (TBA-i) using the method proposed by Vyncke [28] on a 5% trichloroacetic acid extract of the chicken breasts. Briefly, 5.0 mL of the mentioned extract was mixed with 5.0 mL of 0.02 M thiobarbituric acid and incubated at 97 °C for 40 min. The thiobarbituric acid reactive substances were quantified by measuring the absorbance at 532 nm and using 1,1,3,3-tetraethoxy-propane as a standard. The results were expressed as mg malonaldehyde/kg muscle.

#### 2.5.4. Sensory Analysis

Sensory preliminary evaluations of the EOs applied in the vapor phase were performed through a global acceptability test of taste and odor. Two sensory evaluations were carried out. First, individual EOs were assessed, and secondly, the system treated with 1.0% *v*/*v* lactic acid, and the selected EO ternary mixture (C-LA-EO) was evaluated. In both evaluations, an untreated system (C-C) was used as the control. Additionally, for the second evaluation, the system treated with 1.0% *v*/*v* lactic acid (C-LA) and the system treated with the EO ternary mixture (C-EO) were used as controls as well.

The preparation of the samples was carried out as in item 2.5.1. For systems containing EO, paper filter discs were seeded with an individual or ternary mixture of EOs according to the system assayed. After storage at 4 °C for 24 h, the samples (10 g) were cooked individually in an electric oven at 250 °C for 4 min on each side. After that, the samples were divided into 2.5 g portions. The portions were packaged in containers labeled with three-digit numbers, sealed, and stored at room temperature for 5 min to allow the aroma to equilibrate in the HS. After that, 16 consumers (10 women and 6 men) received the samples in a random sequence and evaluated their odor and taste global acceptability through a balanced verbal 9-point hedonic scale (1: dislike very much; 9: like very much) [29]. To achieve this, for each sample, the panelists were required to first smell the sample and rate it and then taste it and rate it. Between samples, they were instructed to drink water. Each assessment was conducted at individual white workstations in comfortable rooms with white walls, devoid of noise and odors.

### 2.6. Statistical Analysis

All assays were performed in triplicate, except the sensory evaluations, which were simplified. In all cases, the averages and standard deviations are reported. An analysis of variance (ANOVA) and the Tukey test were performed to evaluate significant differences among the analyzed parameters. Significance is expressed at a 5% level and tests were performed using Statgraphics Plus version 5.1 (Manugistics, Inc., Rockville, MD, USA).

## 3. Results and Discussion

### 3.1. Assays of Antimicrobial Activity of Essential Oils In Vitro

#### 3.1.1. Screening of Essential Oils’ Antimicrobial Activity

After evaluating the antibacterial effect of EOs, garlic, mustard, and oregano EOs were selected due to their higher activity. In spite of the fact that some of them showed an inhibitory capacity (lemongrass, ginger, and thyme), the three selected EOs presented the biggest inhibition halos (Table 2). The basil, sage, and tarragon EOs did not have any inhibitory effect, while pepper, rosemary, and coriander had a slight effect against *L. innocua.*

Mustard EO was the only one capable of inhibiting all the bacteria assayed. In previous studies, this EO was able to inhibit molds such as *Botrytis cinerea* and *Aspergillus niger*, as well as the following bacteria: *Acinetobacter baumannii*, *Bacillus subtilis*, *Mycobacterium smegmatis*, *Salmonella* Typhimurium, *Staphylococcus aureus*, *Streptococcus pyogenes*, *E. coli*, and *P. aeruginosa* [30,31,32].

Garlic EO showed an important effect in that it totally inhibited *L. innocua*, *P. aeruginosa*, and *L. plantarum*. However, it was not able to inhibit the *P. fluorescens* and *E. coli* strains. Benkeblia [33] tested garlic EO’s effect against *S. aureus*, *Salmonella*, and some molds obtaining inhibitory effects. Similarly, Chao et al. [34] observed its significant effect against *B. cereus*, *S. airrezis*, *S. faecalis*, *Alcaligenes faecalis*, *Enterobacter cloacae*, and *P. aeruginosa*, which was consistent with this test.

Oregano EO was able to inhibit *L. innocua* and *E. coli* but failed with *Pseudomonas* strains and *L. plantarum*. Dobre et al. [35] described its antimicrobial effects against *B. cereus*, *S. aureus*, *S. enteritidis*, and *E. coli*, similar to this research.

#### 3.1.2. Minimal and Fractional Inhibitory Concentrations of Essential Oils in Vapor Phase

The smallest MIC found was that corresponding to mustard EO (0.146 µL/mL of HS) for both bacterial species, showing that, in addition to inhibiting the growth of all the studied microorganisms, it was the most effective EO since its MIC corresponds to 1/8 of the maximum level tested. Oregano EO inhibited *E. coli* growth, showing an MIC of 0.585 µL/mL of HS, which corresponds to a 1/2 dilution of the level assayed for the previous item. Garlic EO inhibited *P. aeruginosa* growth at a concentration of 0.292 µL/mL of HS, which represents 1/4 of the original level.

As for EO binary mixture inhibition assays against *P. aeruginosa*, it was observed that the combined use of mustard and garlic EOs was effective in achieving inhibition. Although different mixtures were found to succeed, the one with the lowest concentration was constituted by 0.036 µL/m of HS each. This represents 1/4 and 1/8 of the MIC of mustard and garlic EO, respectively, and corresponds to a synergistic interaction with a FIC_i_ = 0.37 (Table 3). This is consistent with the results obtained by Aguilar-González et al. [32], who tested the effect of mustard and clove EOs combined in their vapor phase against *B. cinerea* and obtained a FIC_i_ = 0.25. The garlic–oregano and mustard–oregano binary mixtures did not show inhibitory activity against *P. aeruginosa*. This could be due to the fact that oregano EO had no inhibitory effect against this microorganism when used individually. Lambert et al. [36] reported that 1 μL/mL of oregano was the minimum concentration required to inhibit *P. aeruginosa*. Although this value is similar to the maximum concentration used in this study (1.17 μL/mL), given that the composition of an EO depends on a diversity of factors [15], it is suggested that higher doses of the evaluated oregano EO might be necessary to inhibit the growth of *P. aeruginosa*.

Regarding the binary mixture inhibitory assays against the pool of *E. coli*, the unique mixture showing an inhibitory effect was the one constituted by oregano and mustard EOs. By combining these oils, it was possible to reduce their necessary concentrations, since 0.073 µL/mL of HS of mustard EO (1/2 of its MIC) and 0.292 µL/mL of HS of oregano EO (1/2 of its MIC) were able to control the growth of the strains in question, producing an additive interaction in their inhibition (FIC_i_ = 1) (Table 4). As mentioned above, garlic EO had no inhibitory effect against *E. coli* and did not modify the activity of mustard and the oregano EO sub-inhibitory concentrations. Seydim et al. [37] and Du et al. [38] reported similar results when studying the effect of garlic EO in edible films or vapor phase, respectively.

Based on the results obtained, a ternary mixture was selected for subsequent application to chicken breasts. Considering that the interaction between antimicrobial compounds and the food matrix may decrease its effectiveness, the mixture was constituted by 0.073, 0.292, and 0.146 µL/mL of HS of mustard, oregano, and garlic, respectively. As expected, it could be verified that the proposed EO ternary mixture was able to inhibit the growth of *P. aeruginosa* and *E. coli* in vitro.

### 3.2. Chemical Composition of Essential Oils in Vapor Phase

The results of the HS chemical composition of the selected ternary mixture of EOs showed the presence of numerous compounds with recognized antimicrobial and antioxidant activity (Table 5). Among the compounds with high concentrations, p-cymene (30%) was highlighted. The latter did not exhibit significant antibacterial activity when used individually; however, when combined with low concentrations of carvacrol or thymol, it showed a potent antimicrobial effect [39]. In addition, the presence of diallyl disulfide (17.1%), an aromatic compound that gives garlic its characteristic odor, and allyl isothiocyanate (10.2%), a compound responsible for the characteristic odor of mustard with an inhibitory effect against some microorganisms, was detected [40]. Moreover, recognized antimicrobial compounds were present (carvacrol 5.1%, α-pinene 4%, and limonene 4.8%), as well as antioxidant and aromatic substances (α-terpinene 1.9%, 1,8-cineole 0.2%, linalool 0.8%) [41].

### 3.3. Application of Essential Oils on Chicken Breast

#### 3.3.1. Microbiological Analysis

**Figure 1:**
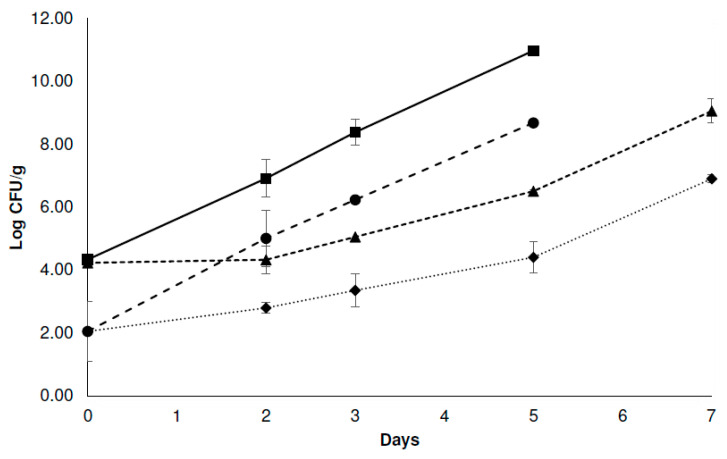
The effect of different treatments against mesophilic aerobic microorganisms in chicken breast meat stored at 4 °C. (■) Control chicken breast free of EOs and lactic acid immersion (C-C); (▲) chicken breast exposed to the EO ternary mixture (C-EO); (●) chicken breast immersed in 1.0% *v*/*v* lactic acid solution (C-LA); (♦) chicken breast immersed in 1.0% *v*/*v* lactic acid solution and exposed to the EO ternary mixture (C-LA-EO). Error bars represent standard deviation.

Figure 1 shows the growth of mesophilic aerobic microorganisms in the different systems studied. The treatments involving 1.0% *v*/*v* lactic acid immersion (C-LA and C-LA-EO) reduced the initial microorganism population, showing a decrease of two log cycles compared to the control samples (C-C). Similar trends have been reported when studying the effect of lactic acid (1.0–5.0%) on the chicken skin microorganism initial counts [6,9]. During the refrigerated storage, an increase in the microorganism population was observed in all the systems. However, differences between them were evident. After 2 days, the C-C systems showed a population of approximately seven log cycles, reaching the maximum limit allowed by the Argentinean Food Code [42]. The other systems remained below this value, being their counts within two and four log cycles lower than the control. Within 5 days, the C-LA system exceeded seven log cycles, and the C-EO system showed counts slightly lower than this value. On the other hand, the system that received the treatment combination (C-LA-EO) achieved a population of 4.4 log cycles and showed values close to 7 log cycles after 7 days of refrigerated storage.

It must be noted that the rate of microbial growth in the system receiving the lactic acid treatment (C-LA) was higher than in those systems containing the EO mixture (C-EO and C-LA-EO). This suggests that the application of the EOs in the vapor phase was more effective than the lactic acid immersion in preserving the microbiological characteristics of the chicken breasts. However, it was also observed that the use of EOs as the only barrier is not enough and that their combination with 1.0% *v*/*v* lactic acid immersion managed to reduce the microbial growth significantly. The effectiveness of EOs applied in their vapor phase has been previously reported. For example, Delcarlo et al. [20] succeeded in controlling the growth of total bacteria in hake fillets through the use of a mixture of oregano and lemongrass EOs applied in their vapor phase and a protective culture. Additionally, Lee et al. [18] reported the antilisterial activities of oregano, thyme, thymol, and cinnamon bark EO gases on the surface of radish sprouts; and Lorenzo-Leal et al. [19] reported that the vapor phases of allspice, thyme, and rosemary EOs exerted inhibitory activity against *L. monocytogenes* and *S*. Typhimurium in alfalfa seeds.

#### 3.3.2. Physicochemical Analysis

**Figure 2:**
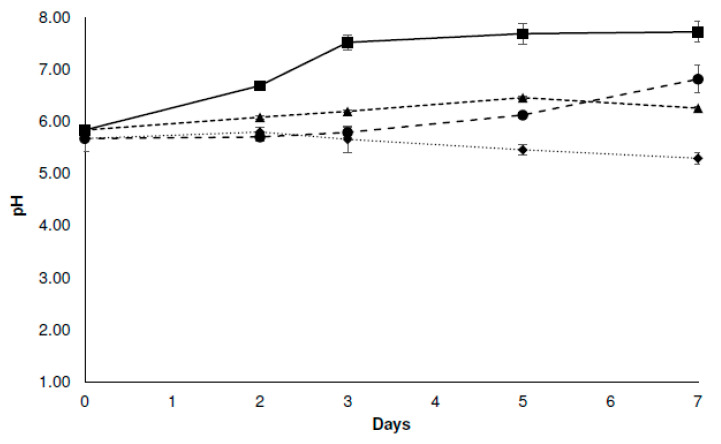
The effect of different treatments on the pH of chicken breast meat stored at 4 °C. (■) Control chicken breast, free of EOs and lactic acid immersion (C-C); (▲) chicken breast exposed to the EO ternary mixture (C-EO); (●) chicken breast immersed in 1.0% *v*/*v* lactic acid solution (C-LA); (♦) chicken breast immersed in 1.0% *v*/*v* lactic acid solution and exposed to the EO ternary mixture (CLA-EO). Error bars represent standard deviation.

The initial pH of the chicken samples was found to be between 5.5 and 6.0, which is considered a normal value for this type of food [43,44]. As expected, the lactic acid immersion caused a slight decrease in the pH (Figure 2). As for the control systems (C-C), it was observed that the pH increased significantly after 3 days of storage and then remained constant at a value approaching 7.5. This increase may be explained by the release of the products of protein metabolism, which is caused by microbial or enzymatic processes [45]. The C-LA system showed a slight increase in pH toward the end of storage, while the C-EO system’s pH remained constant during the 7 days (Figure 2). In contrast, in C-LA-EO system, a slight decrease in its pH value was observed over time. Other studies have reported a decrease in the pH of refrigerated chicken meat with low microbial growth [5].

**Figure 3:**
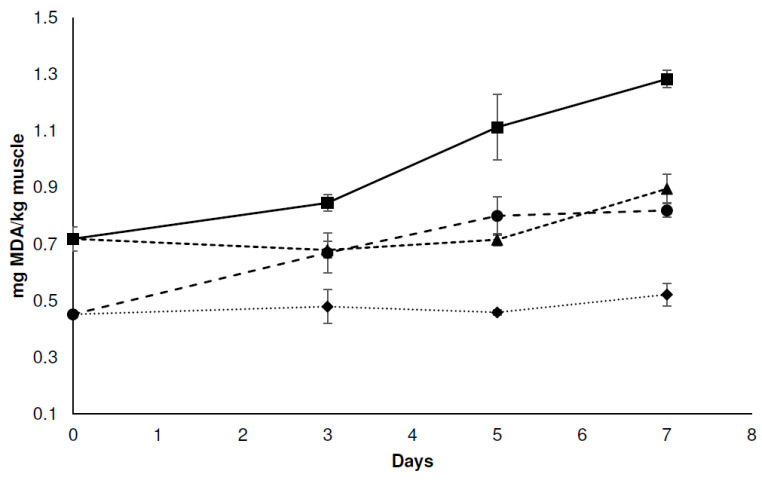
The effect of different treatments on lipid oxidation of chicken breast meat stored at 4 °C. (■) Control chicken breast, free of EOs and lactic acid immersion (C-C); (▲) chicken breast exposed to the EO ternary mixture (C-EO); (●) chicken breast immersed in 1.0% *v*/*v* lactic acid solution (C-LA); (♦) chicken breast immersed in 1.0% *v*/*v* lactic acid solution and exposed to the EO ternary mixture (C-LA-EO). Error bars represent standard deviation.

Lipid oxidation is a critical parameter in the evaluation of meat quality. Although there was a trend of increasing malonaldehyde (MDA) concentration, differences between systems were found. The control system (C-C) showed the highest MDA concentrations during the whole storage period, and a marked increase in this parameter was observed over time (Figure 3). These results are expected in foods packaged with air chambers. The increase in MDA may be due to microbial enzymes acting on food lipids, which generates free fatty acids that are highly susceptible to oxidation [46]. The superior microbial growth observed in the C-C system would explain the results obtained when lipid oxidation was evaluated. The system that received the lactic acid immersion (C-LA) also showed an increase in lipid oxidation until the fifth day of storage, after which the value remained constant (Figure 3). It must be noted that the values observed for this system were always below the control values. Organic acids are able to combine with oxygen compounds and sequester trace metals, thus acting as antioxidants [47]. In the case of the systems exposed to the EOs (C-EO), the concentration of MDA remained constant for 5 days and then increased slightly (Figure 3). EOs contain phenolic compounds with known antioxidant activity [41]. The obtained results are in agreement with those reported by Fratianni et al. [48], who evaluated the effect of thyme and balsam EOs on refrigerated chicken and were able to verify their antioxidant activity. Systems containing the combination of preservation factors (C-LA-EO) were the most efficient in protecting the chicken meat from lipid oxidation. In this case, the value remained constant throughout storage (Figure 3).

#### 3.3.3. Sensory Analysis

**Figure 4:**
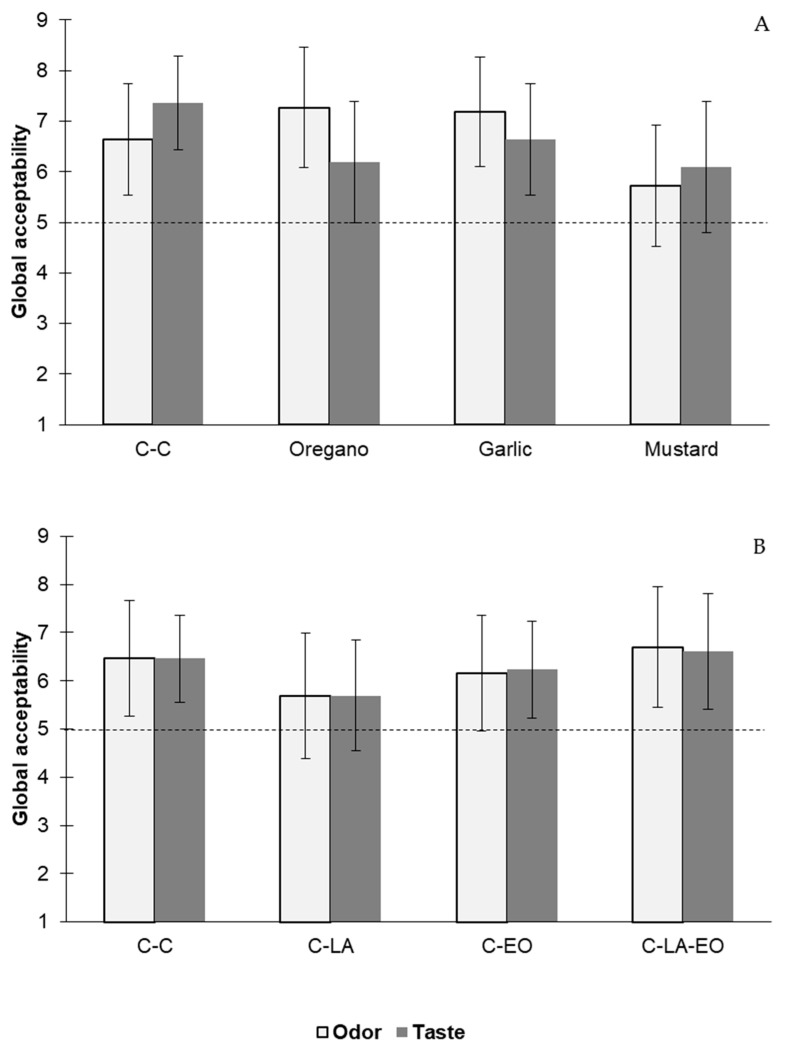
The acceptability of odor and taste in cooked chicken breast meat treated with individual EOs of oregano, garlic, and mustard (**A**) and treated with lactic acid and the ternary mixture of EOs (**B**). Control chicken breast, free of EOs and lactic acid immersion (C-C). Chicken breast exposed to the EO ternary mixture (C-EO). Chicken breast immersed in 1.0% *v*/*v* lactic acid solution (C-LA). Chicken breast immersed in 1.0% *v*/*v* lactic acid solution and exposed to the EO ternary mixture (C-LA-EO). Error bars represent standard deviation.

Figure 4 shows the results of the sensory analysis of the individual EOs (Panel A) and the combination of lactic acid and the EO ternary mixture (Panel B). For all treatments, there were no significant differences between the systems compared to the control in terms of either of the two parameters evaluated. For both parameters, all the systems exceeded a value of five, which was assigned to “neither like nor dislike”. This indicates that the application to the chicken samples of each EO individually and the ternary mixture alone or in combination with lactic acid did not negatively affect the odor or taste acceptability of the chicken breasts. The C-LA-EO system showed scores of 6.6 (like moderately) for both the parameters assayed, which were similar to the control chicken meat. In other studies, similar observations have been made, such as that rosemary and Chinese mahogany EOs did not negatively affect the acceptance of refrigerated chicken meat [49].

## 4. Conclusions

The mustard, oregano, and garlic EOs evaluated in their vapor phase in vitro exerted strong inhibitory activity against the evaluated microorganisms. The binary mixtures of mustard–garlic and mustard–oregano EOs were revealed to be synergistic against *P. aeruginosa* and additive against *E. coli*, respectively. The vapor phase application of an EO ternary mixture (0.073, 0.292, and 0.146 µL/mL of HS of mustard, oregano, and garlic, respectively) in the chicken breasts improved some microbiological and physicochemical characteristics of the food during refrigerated storage. The combination of the EO ternary mixture and 1.0% *v*/*v* lactic acid immersion reduced the total mesophilic microorganisms’ population as well as lipid oxidation for 7 days. In addition, the samples thus treated were positively accepted by consumers. The use of EOs in their vapor phase together with lactic acid immersion proved to be an effective alternative for increasing chicken breast shelf life through the use of natural antimicrobials in the context of hurdle technology.

## Figures and Tables

**Table 1:** Binary mixtures’ concentrations of EOs.

Binary Mixture	EO A Concentration	EO B Concentration
1	MIC *	-
2	-	MIC
3	1/2 MIC	1/2 MIC
4	1/2 MIC	1/4 MIC
5	1/4 MIC	1/2 MIC
6	1/4 MIC	1/4 MIC
7	1/4 MIC	1/8 MIC
8	1/8 MIC	1/4 MIC

* Minimal inhibitory concentration against a specific microorganism.

**Table 2:** Diameter (mm) of the inhibition halos produced by EOs in their vapor phase.

Microorganisms	Essential Oils					
	Lemongrass	Ginger	Garlic	Mustard	Oregano	Thyme
*L. innocua*	15.0 ± 1.0	_	>52.0	>52.0	40.0 ± 2.0	30.0 ± 1.0
*P. aeruginosa*	21.5 ± 0.5	_	>52.0	>52.0	_	_
*P. fluorescens*	_	_	_	>52.0	_	_
*L. plantarum*	11.5 ± 0.5	21.5 ± 0.5	>52.0	>52.0	_	_
*E. coli* ATCC 35218	_	_	_	>52.0	>52.0	23.0 ± 1.0
*E. coli* ATCC 25922	_	_	_	>52.0	40.0 ± 1.0	31.0 ± 1.0

“_” Without inhibition.

**Table 3:** The effect of binary mixtures of mustard and garlic EOs in their vapor phase against *P. aeruginosa*.

EO Concentration (MIC Fraction)			FIC_i_
Mustard	Garlic	*P. aeruginosa*	
1	0	No growth	
0	1	No growth	
1/2	1/2	No growth	1.0
1/2	1/4	No growth	0.75
1/4	1/2	No growth	0.75
1/4	1/4	No growth	0.50
1/4	1/8	No growth	0.37
1/8	1/4	Growth	

Mustard MIC: 0.146 µL/mL of HS; garlic MIC: 0.292 µL/mL of HS.

**Table 4:** The effect of binary mixtures of mustard and oregano EOs in their vapor phase against *E. coli*.

EO Concentration (MIC Fraction)			FIC_i_
Mustard	Oregano	*E. coli*	
1	0	No growth	
0	1	No growth	
1/2	1/2	No growth	1.0
1/2	1/4	Growth	
1/4	1/2	Growth	

Mustard MIC: 0.146 µL/mL of HS; oregano MIC: 0.585 µL/mL of HS.

**Table 5:** Headspace chemical composition of the selected EO ternary mixture.

Compound	%
diallyl sulfide	2.9
allyl isothiocyanate	10.2
disulfide, methyl-2-propenyl	0.6
α-pinene	4.0
camphene	0.5
myrcene + β-pinene	6.5
α-phellandrene	0.5
α-terpinene	1.9
p-cymene	30.1
limonene	4.8
1,8-cineole	0.2
γ-terpinene	9.5
diallyl disulfide	17.1
linalool	0.8
trisulfide, methyl-2-propenyl	0.4
carvacrol	5.1
trisulfide, di-2-propenyl	1.5
caryophyllene oxide	1.2
total	97.8

## Data Availability

The data presented in this study are available on request from the corresponding author.
